# Industrial exoskeletons from bench to field: Human-machine interface and user experience in occupational settings and tasks

**DOI:** 10.3389/fpubh.2022.1039680

**Published:** 2022-11-21

**Authors:** Antonio Baldassarre, Lucrezia Ginevra Lulli, Filippo Cavallo, Laura Fiorini, Antonella Mariniello, Nicola Mucci, Giulio Arcangeli

**Affiliations:** ^1^Department of Experimental and Clinical Medicine, University of Florence, Florence, Italy; ^2^Department of Industrial Engineering, University of Florence, Florence, Italy; ^3^School of Occupational Medicine, University of Florence, Florence, Italy

**Keywords:** industrial exoskeleton, occupational medicine, work-related musculoskeletal disorders, user experience, Human-Robot Cooperation, ergonomics, occupational safety and health (OSH), personal protection equipment (PPE)

## Abstract

**Objective:**

Work-related musculoskeletal disorders (WRMSDs) are considered nowadays the most serious issue in the Occupational Health and Safety field and industrial exoskeletons appear to be a new approach to addressing this medical burden. A systematic review has been carried out to analyze the real-life data of the application of exoskeletons in work settings considering the subjective responses of workers.

**Methods:**

The review was registered on PROSPERO. The literature search and its report have been performed following the PRISMA guidelines. A comprehensive literature search was performed in PubMed, EMBASE, Web of Science, and Scopus.

**Results:**

Twenty-four original studies were included in the literature review; 42% of the papers retrieved included automobilist industry workers, 17% of the studies evaluated the use of exoskeletons in logistic facilities, and 17% of articles involved healthcare. The remaining six papers recruited farmers, plasterers, wasting collectors, construction workers, and other workmen. All the papers selected tested the use of passive exoskeletons, supporting upper arms or back. Usability, perceived comfort, perceived exertion and fatigue, acceptability and intention to use, occupational safety and health, and job performance and productivity were the main topic analyzed.

**Conclusion:**

Exoskeletons are not a fix-all technology, neither for workers nor for job tasks; they tend to show more of their potential in static activities, while in dynamic tasks, they can obstacle regular job performance. Comfort and easiness of use are the key factors influencing the user's experience. More research is needed to determine the most effective and safe ways to implement exoskeleton use in occupational settings.

**Systematic review registration:**

https://www.crd.york.ac.uk/prospero/display_record.php?RecordID=275728, identifier CRD42021275728.

## Introduction

The European Agency for Safety and Health at Work (EU-OSHA) has recently reported that around 60% of all workers with a work-related health problem, identify work-related musculoskeletal disorders (WRMSDs) as their most serious issue, representing also one of the most common accidents. According to EU-OSHA 2019 report (1), roughly three out of every five workers in the European Union report WRMSD complaints. The most common types of WRMSDs reported by workers are backache and muscular pains in the upper limbs, especially in the construction, water supply, agriculture, forestry, and fishing sectors. Women report slightly more WRMSDs than men. WRMSDs prevalence is higher among older workers while decreasing with educational level (2), considering also that a higher level of education almost always corresponds to a lower chance of carrying out physically demanding jobs (3, 4). WRMSDs affect the general health of workers, that tend to be absent from work more often than others, resulting in a very high impact of WRMSDs in economic terms (5). Analysis of the phenomenon identified a significant relationship between self-reported WRMSDs and some physical risk factors; the advent of industry 4.0 meant a slight decrease in the prevalence of most physical risks, except for working with computers, laptops, and smartphones, while the back of the coin has resulted in a significant relationship between self-reported WRMSDs and organizational and psychological risk factors since more than half of workers complain about work-related stress (4). Occupational Health and Safety Management Systems (OHSMS) have forward-looking sustainable solutions to preserve employees' health and physical wellbeing (6–9). In this sense, workplace ergonomics can be considered a major point to achieve this goal (10). Improving workplace ergonomics should be treated as a continuous process, which systematically identifies and effectively reduces the level of workers' exposure to the risk factors known to cause WRMSDs. Until today, ergonomics improvement processes have been based on a continuous improvement model such as the quality (ISO 9001), environmental (ISO 14001), and safety (OHSAS 18001) models, without forgetting the ISO 12100, regarding the safety of machinery, and including general principles for design, aimed at reducing risk. Each of these management system models provides a common and familiar set of steps for managing environmental and safety risks, including WRMSDs risks. The recently introduced ISO 45001 (Safety Management System standard) provides a new, and soon-to-be common, model that can be used as an effective system for managing ergonomics. As result, most employers have implemented one or several preventive measures: rotation of tasks to reduce repetitive movements or physical strain, encouraging regular breaks for people in uncomfortable or static postures including prolonged sitting/provision of ergonomic equipment or equipment to help with lifting or moving, promoting healthy lifestyles (11). Newly developed Information Technology systems for ergonomics assessment help companies to evaluate how ergonomic their workstations are (12–16). At the same time, they enable qualification profiles to be drawn up for the assignment of physically impaired employees in line with their abilities and minding principles based also on the WHO International Classification of Functioning, Disability and Health (ICF) (17). Also, innovative solutions are provided by Human-Robot Cooperation (HRC) and the possibility to equip employees with wearable computing systems and devices in production facilities (18–24). In this scenario, the exoskeletons undoubtedly represent the state-of-the-art technological evolution made available to health and safety, providing useful opportunities also in the framework of Industry 4.0 and human-machine cooperation (25). The first research on exoskeletons dates to the early 1970s with projects undertaken for military purposes, aiming to produce armors that would enhance the user's strength, but colliding with technological limits in terms of sensors, structures, and actuators, resulting in systems that were too heavy and inefficient (26). Subsequently, the patent designs for exoskeletons have taken off in the past decade in the industrial sector with improved designs and materials, much more efficient and resistant to mechanical stress thanks to research and development (R&D). In addition, exoskeletons have been used in medical rehabilitation or occupational therapy, for example, to enable victims of spinal injuries to walk again (27–30). In recent decades, research has focused considerably on the development of this technology and exoskeletons have entered various fields and sectors, from military (31) to industrial and more generally manual work (32–34). Exoskeletons appear to be a new approach to addressing the issue of WRMSDs (35, 36), especially when other organizational interventions in workplaces are not feasible. Industrial exoskeletons are designed to mechanically assist workers during strenuous and physically demanding tasks, which include the lifting of loads or overhead work. Exoskeletons may light the load or enhance human strength, and this is of particular interest when it is not possible to improve workplace design and layout, as in temporary workplaces.

Industrial exoskeletons can be classified by kinematic structure and type of actuation. Concerning kinematics, rigid-structure devices can be classified as anthropomorphic and nonanthropomorphic devices, whereas soft exosuits do not present any kinematic structure. Moreover, the structure can range from full-body devices to devices that assist only one joint, thus making them potentially tailorable. Actuation types include passive, semi-active, and active systems. The passive exoskeletons do not have actuators or electronic components, such as transducers or controllers, but the force necessary to assist the actions performed by the user is released by exploiting the elasticity of the materials (i.e., torsion springs or pistons). The advantages offered by this type are in the lightness, economy, and simplicity of redesign, unlike the powered (semi-active and active) ones. Due to those peculiar characteristics, exoskeletons work in tandem with the wearer, lightening the load and increasing the physical capabilities of the wearer, as well as solving potential ergonomic issues of holding and working with tools.

Several experimental studies and literature reviews have been conducted to analyze the biomechanical effects of the available exoskeletons on users. Three recent systematic reviews analyzed the effects of industrial exoskeletons on exertion, muscle activity, and partially on user experience (37–39). They found a significant reduction in the user's acute physical stress and strain in the target area (40) and mean values of metabolic and cardiorespiratory parameters (41); also user endurance seemed to improve with the exoskeleton but performance declined in agility tasks (37). Only the review of Kermavnar et al. (37) included in the analysis aspects linked to user experience and satisfaction, which was moderate, and efficacy was rated from low to modest. However, most of the included studies recruit healthy and young volunteers, with no previous working experience and test the exoskeletons in controlled environments performing standardized and simple movements. This kind of lab environment is actually very far from real working settings, where job dynamics are complex, and workers are a heterogenous population. The redistribution of stress to different parts of the body can have effects on workers' health indeed and can play a huge role in the overall acceptance of exoskeletons in the workplace. Designing equipment that is user/worker centered is fundamental to make exoskeletons accepted, according to the idea that machines should adapt to workers and that a wearable product must not only be safe, comfortable, useful, and usable but also just as importantly, must be desirable to the end user (40, 41). Recently, data on the subjective experience of workers wearing the exoskeletons in working environments or performing real job tasks are slowly emerging. Nevertheless, evidence of the effectiveness of industrial exoskeletons in real occupational settings is missing.

Our review aimed at filling the gap in the literature about the worker's experience using the exoskeleton in real working tasks, since studies performed in the lab may have a limited value, not considering the complexity of a work environment as well as the experience of workers, who are the final users of industrial exoskeletons. The purpose of this systematic review is to analyze real-life data on the use of exoskeletons in work settings. In particular, this paper aims to outline a picture of the possible impact, benefits, and criticalities of exoskeletons in the workplace by reviewing and analyzing the subjective responses of workers.

## Materials and methods

### Literature research and data collection

This systematic review was registered on the PROSPERO register at number CRD42021275728 on September 30th 2021. The literature search and its report have been performed following the PRISMA guidelines (42). A comprehensive literature search was performed in PubMed, EMBASE, Web of Science, and Scopus. The search strategy combined free text and controlled vocabulary, using the keywords “exoskeleton” and “worker”. No limits were applied to the search regarding the period of publication and the study design; only papers written in English were deemed eligible for the selection. The details of the search strategy for each database are listed in Supplementary material. Moreover, manual research was performed to screen the bibliographic references of the most relevant papers selected. The research was based on the modified PICO scheme, the SPICE framework (43):

#### Setting

Working environment or lab setting in which working tasks are performed.

#### Perspective

Workers of all types.

#### Intervention

Use of any kind of exoskeleton in an occupational setting (field study/laboratory study reproducing working tasks).

#### Comparison

When present, the use of no exoskeleton for the performance of the same tasks.

#### Evaluation metrics

User's subjective experience/perception/compliance/usability of the device.

A first screening of the results was performed by two independent reviewers (LGL and AM) through titles and abstracts of the reports identified. A further selection was made by analyzing the full text of the articles. The judgment about the inclusion of each paper was performed separately by the investigators; disagreements were solved by the discussion with a third reviewer (AB).

Data were manually extracted in a chart jointly developed by the authors, including relevant data from the papers retrieved.

### Inclusion criteria

The inclusion criteria followed the SPICE scheme mentioned above; we included articles focusing on the application of exoskeletons in the occupational field. We selected papers considering the use of exoskeletons by workers acting in real occupational tasks, both in the field and in the lab. The studies selected took into account the subjective perspectives of workers wearing the exoskeletons regarding comfort, usability, and personal experience.

### Exclusion criteria

Articles written in languages other than English were excluded. Studies analyzing stereotyped tasks which did not reproduce a real working task, or not considering the subjective experience of the participants were also excluded. We also excluded articles that did not conduct experiments on real workers. Also, review articles were not included in the literature synthesis but were discussed in other paragraphs.

### Quality assessment

The quality assessment was performed using the Mixed Methods Appraisal Tool (MMAT) (44, 45) which is designed for the appraisal stage of systematic mixed studies reviews, i.e., reviews that include qualitative, quantitative, and mixed methods studies, thus, it was suitable for our review. The full presentation of the ratings is reported in Supplementary Table S1.

## Results

**Figure 1:**
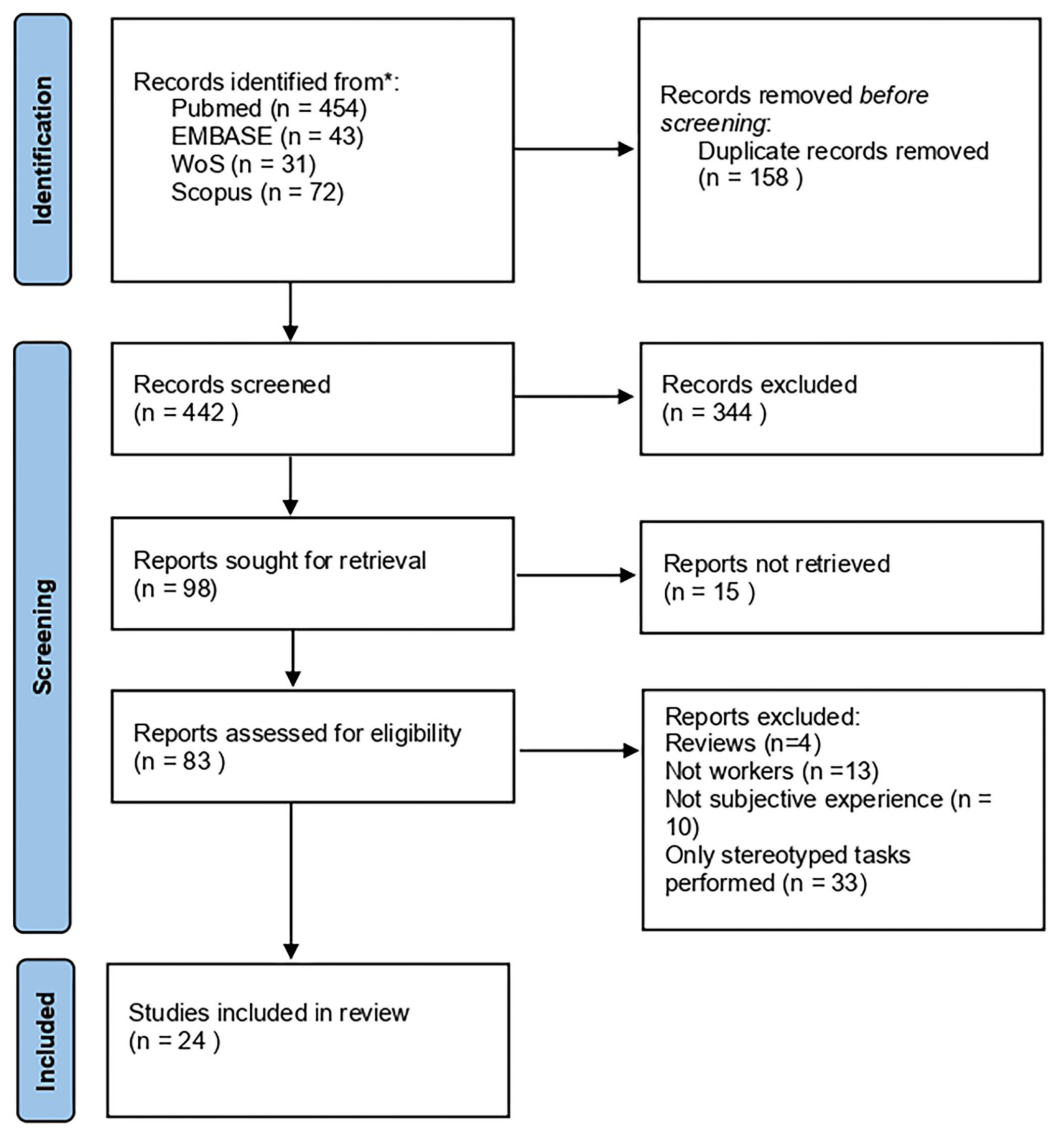
PRISMA 2020 flow-chart (42).

The online search retrieved a total of 600 papers. The selection process is shown in Figure 1.

At the end of the selection, 24 original studies were included in the literature review. Most (sixteen) of the selected articles have a cross-sectional design, administering validated and not validated questionnaires at the end of an experimental period in which the workers perform their job wearing the exoskeleton. Four studies adopted a mixed-method approach and 1 was purely qualitative; the experiment performed is similar to that of a cross-over study: the workers wear the exoskeleton for a period and then evaluate their experience. Three articles described prospective cohort studies of two working populations studied for several months. Fourteen studies were published in Europe, 8 in North America, 1 in Iran, and 1 in Korea. The overall quality of the studies, evaluated with the MMAT tool, ranges from 20 to 80%: 5 studies scored 80%, 13 scored 60%, and 6 scored 40%. The details of the quality appraisal are included in Supplementary material S2.

**Figure 2:**
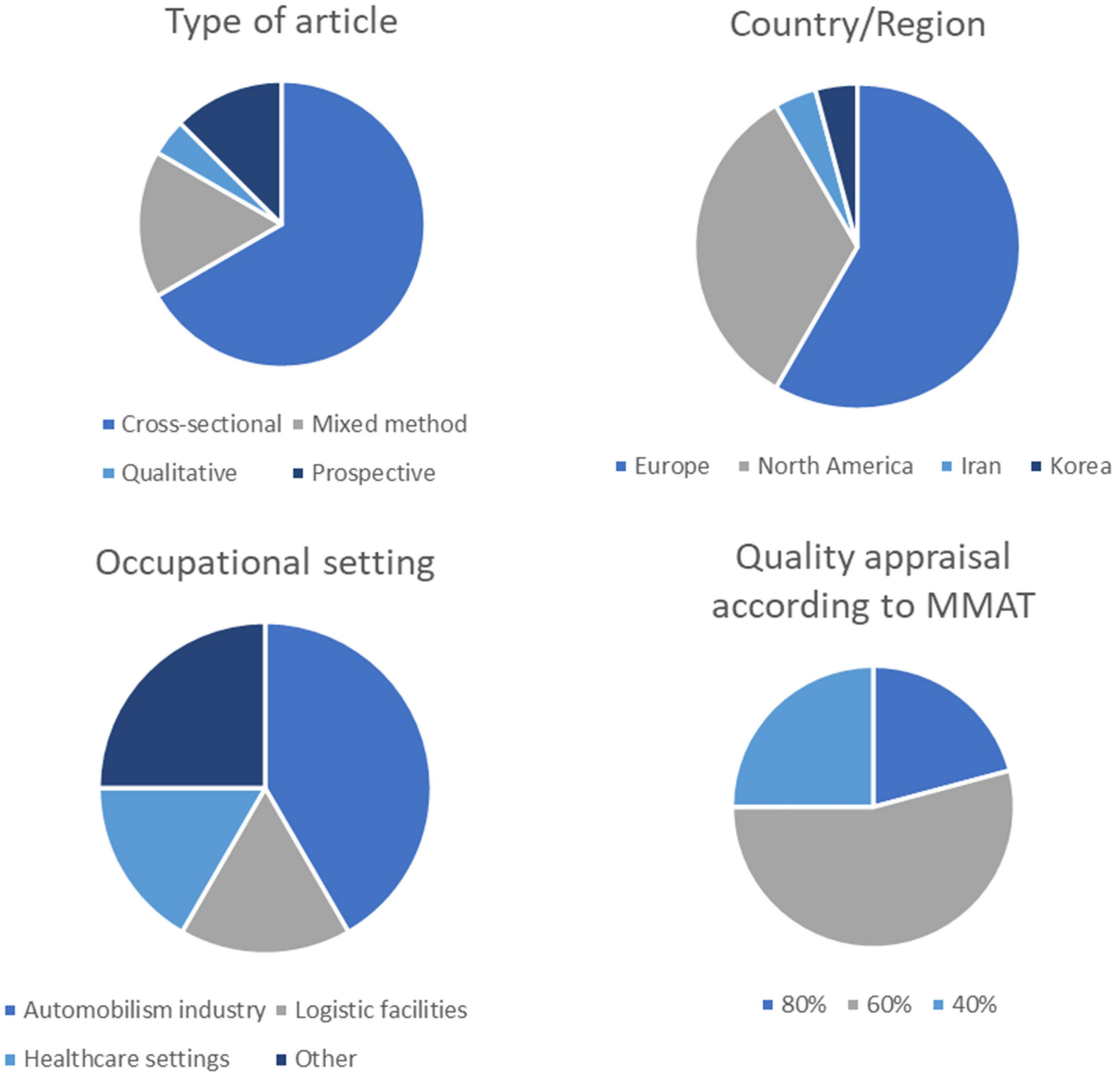
Summary of the selected studies in terms of type of study, country/region, occupational setting and quality appraisal.

42% (*n* = 10) of the selected articles included automobile industry workers, who work in the assembly lines of vehicles; 17% (*n* = 4) of the studies evaluated the use of exoskeletons in logistic facilities, 17% (*n* = 4) of the articles involved healthcare workers such as surgery team members and professional caregivers. The remaining 6 papers (25%) recruited farmers, plasterers, wasting collectors, construction workers, and other workmen. Table 1 includes the selected studies of the review and Figure 2 summarizes the main characteristics of the selected articles.

**Table 1:** Selected studies of the review.

**Author**	**Country**	**Type of study**	**Model of exoskeleton**	**Participants**	**Length of observation**	**Task performed**	**Outcomes**	**Scale used**
Amandels (46)	Belgium	Experimental cross-over	Back support; LAEVO	9 press and shear workers	3 weeks of use/30 minutes experiment	Task involving far reaching with bending over to place or collect items.	Comfort; user's experience	Discomfort Likert-scale; structured user-experience questionnaire.
Antwi-Aftari et al. (47)	UK	Experimental cross-over study	Back support	10 Construction workers	One experiment	Manual repetitive handling tasks (e.g., lifting, carrying, pulling, pushing)	Usability; comfort.	SUS scale, perceived discomfort scale; LPP scale for Perceived musculoskeletal pressure.
Cha et al. (48)	USA	Mixed methods study	Upper limb support; Levitate AIRFRAMe	14 surgical team members	10 min experiment	Surgical tasks of laparoscopic surgery	Usability	Focus groups interview, SUS score
Chae et al. (49)	Korea	Mixed methods study	Lower limbs support; Hyundai chairless exoskeleton (CEX).	27 workers in the automotive assembly or drilling tasks	One experiment	Fixing and dismantling screws	Comfort; usability	Ad hoc questionnaire, interviews, Likert scales.
Daratany and Taveira (50)	USA	Quasi experimental cross-over study	Upper limb support; Ekso Vest	8 automotive assembly workers	8 min experiment	Low force overhead tasks	Perceived exertion; comfort; usability	Ad hoc questionnaires; Technology Acceptance Model (TAM)
De Bock et al. (51)	Belgium	Experimental cross-over study	Upper limb support; ShoulderX, Skelex	4 industrial workers of a distribution center	One experiment	Ground-level order picking, forklift order picking	Comfort; perceived exertion; usability	Body part discomfort scale and the NASA-TLX (task load index) questionnaire
De Vries et al. (52)	Netherlands	Experimental cross-over study	Upper limb support; Skelex 360	Plastering workers	One experiment	Applying gypsum, screeding, finishing with a spatula	Perceived exertion	Borg scale (RPE)
Flor et al. (53)	Portugal	Experimental cross-over study	Back support; LAEVO	23 automotive assembly workers	4 weeks	Grabbing pressed parts and placing in containers; construction, maintenance, and manualing tooling of molds (non cyclic tasks involving trunk flexion)	Job performance; comfort; perceived exertion; usability; acceptability; usability.	Single Use Question (SEQ); Borg CR10 scale for perceived effort; 7 points Likert scale for discomfort; Post study system usability questionnaire (PSSUQ); 7 point Likert scales for other variables
Gilotta et al. (54)	Italy	Case Series	Upper limb support; Levitate Personal Lift Assist	automotive assembly workers	one experiment	sealing and moving weights	Usability; acceptability	Usability metrics questionnaire; TAM2 questionnaire; qualitative approach
Hensel and Keil (55)	Germany	Experimental cross-over study	Back support; LAEVO	30 automobile industry workers	4 weeks observation	automobile assembly tasks statics and dynamic	discomfort; usability; intention to use	Body part discomfort scale, UMUX lite tool
Hwang et al. (56)	USA	Experimental cross-over study	Back support; FLx ErgoSkeleton, V22 ErgoSkeleton, and Laevo V2.5	20 professional caregivers	One experiment	Patient transfer in the lab	Usability	SUS -System Usability Scale
Kim et al. (57)	USA	Prospective cohort study	Upper limb support; EksoVest	Automotive assembly operators	18 months obeservation	Overhead tasks in the automotive assembly line	Perceived strain; comfort.	Psychological climate and effort measures questionnaire; Cornell Musculoskeletal Discomfort Questionnaire (CMDQ)
Kim et al. (58)	USA	Prospective cohort study with mixed methods design	Upper limb support; EksoVest	65 assembly workers	18 months obeservation	Overhead assembly work	Comfort; safety; job performance; acceptability	Ad hoc questionnaires, open questions
Liu et al. (59)	USA	Experimental cross-over	Upper limb support; Levitate AirFrame	7 general surgery residents and attendings	5–15 min experiment/whole day	Surgical activities.	Perceived exertion.	Ad hoc questionnaire a
Motmans et al. (60)	Belgium	Experimental cross-over	Back support; LAEVO	10 order pickers	1.5 h experiment with and without the exoskeleton	Manual picking	User's experience	Ad hoc questionnaires
Moyon et al. (61)	France	Experimental cross-over study	Upper limb support; Skelex	9 sanders	one experiment	Sanding with three or four different papers, coating, polishing, painting.	Usability	Global satisfaction scale Usability engineering scale
Omoniyi et al. (62)	Canada	Cross-sectional qualitative study	Back support; LAEVO	10 Farmers	One experiment	Farming activities	Safety; job performance	Face-to-face, semistructured interviews
Pacifico et al. (63)	Italy	Case-series study	Upper limb support; MATE	7 enclosures production workers	2 h experiment	Mounting, dismounting, and hanging panels in a panting area	Usability; acceptability	The Local Perceived Exertion (LPE) test; ad-hoc usability questionnaire; Technology Acceptance Model (TAM)
Siedl and Mara (64)	Austria	Experimental cross-over	Back support; LAEVO	31 logistic workers	30 min experiment	Logistic activities (receipt of goods from suppliers, order picking jobs, packaging, internal transport)	Usability; job performance; intention to use; perceived exertion	Task-Specific Self-Efficacy (TSSE) Intention to Use (ITU); German Technology Usage Inventory
Smets (65)	USA	Prospective cohort study	Uper limb support	8/10/4 automotive assembly operators	Few minutes/4 h/3 months observation	Overhead tasks at assembly line	Comfort.	Functionality Questionnaire; Fit & Functionality Questionnaire
Spada et al. (66)	Italy	Mixed methods study	Upper limb support; IUVO	18 assembly workers of an automotive plant	One experiment	assembly tasks including holding posture, precision tasks, manual handling tasks	Usability; acceptability	Semistructured interview; the Borg rating of perceived exertion scale.
Turja et al. (67)	Finland	Mixed methods study	Back support; LAEVO	16/7 Nurses	One experiment/one week	Geriatric care (assisting a patient out and into a wheelchair, eating, and toileting)	Usability; acceptability	Semistructured interviews; Ad hoc questionnaires
Winter et al. (68)	Germany	Experimental cross-over study	Back support	8 logistic workers	2 h experiment	Picking of goods	Perceived exertion; comfort	Ad hoc questionnaires
Ziaei et al. (69)	Iran	Experimental cross-over study	Back support; Ergo-Vest	20 wasitng collector	8-hour shift	Wasting collecting activities	Comfort; perceived exertion; usability.	Custom-made questionnaires; Borg's Rating Perceived Exertion (RPE) scale; System Usability Scale (SUS)

### Models of exoskeletons tested

All the papers selected tested the use of passive exoskeletons. Two main types of exoskeletons were worn by the workers: exoskeletons supporting the upper limbs and exoskeletons supporting the back. The types of exoskeletons were chosen according to the job performed. Only one study provided results of usability on lower limbs supporting exoskeleton (Hyundai CEX chair exoskeleton) (49).

Table 2 includes the models of the upper limb exoskeleton used in the selected studies. The weight of the exoskeletons ranges from about 5 kg (ShoulderX) to 2 kg (Levitate AirFrame). The exoskeletons are designed to reduce the load on the shoulders during work at shoulder height or higher and, as a result, prevent shoulder injuries. Different models can provide support when the arms are forward flexed or adducted. The exoskeleton is worn as an upper body suit, and it is connected to the trunk. Most exoskeletons are available in two different sizes and can be further adjustable. Also, the support provided in some models can be regulated by the user.

**Table 2:** Upper limb support exoskeletons tested in the selected studies.

**Name of the exoskeleton**	**Part of body supported**	**Type of job performed**
Muscular Aid Technology exoskeleton (MATE) (69)	Shoulders	Enclosures production line: mounting, dismounting, and hanging panels in a panting area
ShoulderX (V2, SuitX, Emeryville, United States) (59)	Shoulders	Logistic setting: picking
Skelex (59, 63, 70)	Shoulders/upper body	Logistic setting: picking; Plastering activities Sandering activities
Levitate AirFrame (55, 58, 66)	Upper body	Surgical activities; Automotive plant assembly activities
IUVO (48)	Upper limb	Automotive plant assembly activities
EksoVest (51, 62, 65)	Upper limb	Automotive plant assembly activities

Table 3 shows the exoskeleton models for back support tested in the selected papers. The weight of the back support exoskeleton ranges from 0.9 kg (ErgoVest) to over 2 kg (Laevo). Nevertheless, the Laevo model was the most used. The exoskeletons came in different sizes and can be further adjusted. The back support exoskeletons are supposed to support lifting and forward bending activities and heavy workload manipulation at the workplace. The devices act by transferring a portion of the force from the spinal column to the shoulders, pelvis, and legs. In a study, the exoskeleton tested was a novel model aimed to reduce lumbar back loadings during awkward postures (i.e., stoop and squat postures) and during lifting/lowering/carrying in repetitive manual handling activities (47).

**Table 3:** Back support exoskeletons tested in the selected studies.

**Name of the exoskeleton**	**Part of body supported**	**Type of job performed**
FLx ErgoSkeleton (50)	Back	Patient transfer
V22 ErgoSkeleton (50)	Back	Patient transfer
Laevo (50, 53, 56, 57, 60, 61, 67, 68)	Back	Patient transfer; Logistic activities involving manual picking; Press and shear activities; Automotive assembly tasks Farming activities;
ErgoVest (64)	Back	Wasting collecting activities

### Scale used

The selected studies used several validated tools, *ad hoc*- not validated - questionnaires and qualitative approaches to investigate the worker's experience wearing the exoskeleton. Usability was measured with the SUS – System Usability Scale (71), in its original form and other version adapted by authors to the exoskeleton experience. Usability and user experience were also measured with the Post-Study System Usability Questionnaire (PSSUQ) (72), with the Usability metrics questionnaire (73), and with the scale by Laugwitz et al. (74). Global satisfaction was measured with the tool by Nielsen (75). The Technology Acceptance Model (TAM) questionnaire was used to assess the perceived efficacy, usability, and acceptance of the device (76). The German Technology Usage Inventory assessed the Intention to Use and usefulness (77). Other validated tools adopted in the studies are the Usability Metric for User Experience lite (UMUX) (78) and the scale to evaluate Task-Specific Self-Efficacy (TSSE) (79, 80). Exertion, physical load, and comfort were analyzed with NASA-TLX (task load index) questionnaire (81), Borg's Rating Perceived Exertion (RPE) scale (82), Local Perceived Pressure (LPP) scale (83), and Cornell Musculoskeletal Discomfort Questionnaire (CMDQ) (84). Qualitative approaches included focus groups and semistructured interviews (48, 49, 54, 58, 62, 66, 67).

### Usability

Eighteen papers (75%) investigated the concept of usability of the exoskeleton worn by workers. The scale used to evaluate usability was heterogenous, the most used was SUS (System Usability Scale), which was adopted by 4 studies. The scales used by each study are reported in Table 1. For all the exoskeletons tested, the usability was judged from medium to high. Key features of the usability of an assistive wearable device are the easiness of use, simple design, and minimal effort to learn (56). Also, the device should be effective, flexible, intuitive, and easy to use (54). The usability is strictly connected to lightweightness and easiness to wear (47, 50, 67). Usability decreases as body discomfort and frustration intervene in the task (51). In surgery settings (59), worker role and specific operation were key factors identified for successful implementation and improved usability: the constant movement and the need to take on and off the device to speak with the patients required by the attending surgeon prevent this figure to benefit the most from the device. However, the surgical team confirmed that this technology can be relevant to reduce physical load and identified the time needed for donning/doffing the exoskeleton as a key term for usability. In the automotive tasks, the study by Chae et al. (49) underlined the importance of wearability, in the part of fastening and adjusting the harness and finding the right position. Stability and convenience were the most important factors related to overall wearing satisfaction. In the study by Hensel and Keil (55), workers reported moderate-high levels of usability, which suggests a good level of support to reduce physical demands, though this aspect of usability decreased from the start to the end of the trial.

### Perceived comfort

Thirteen studies (54%) addressed the perceived comfort and discomfort of the workers wearing the exoskeletons. There was no homogeneity in the scale used to measure physical comfort or discomfort. The most used scale was a Likert scale measuring discomfort, adopted by four studies. The physical comfort of the device is mainly expressed in the direct interface between the exoskeleton and the body. In logistic workers, participants reported the highest discomfort in the shoulder regions where the rigid frame of the exoskeleton and the user's body interacted, or body parts onto which the exoskeletons were attached. These aspects influenced the score of usability (51). In the automotive plants, the physical discomfort on the lower back perceived by workers wearing back support exoskeletons and working in static positions, decreases significantly over time, thus suggesting a potential for the devices to reduce physical demands on the lower back. On the contrary, this improvement in comfort was not found for the dynamic workstation, where workers tended to report increasing uneasiness during that time (55). Similar findings were found in the construction setting, where workers working with increased lifting loads rated a reduced level of perceived discomfort in their lower back while using a back support exoskeleton system (47). In automotive settings, the body region that was found more involved in the perceived discomfort was the chest. The discomfort was attributable to the chest pad of the back support exoskeleton, which caused pain in particular in dynamic workload situations (55). Other body regions involved were the upper back, chest, hips, and thighs due to friction, pressure, and heath. For certain tasks that involved trunk rotation, the rigid bars of the back support exoskeleton made pressure on the thoracic region causing pain (53).

Thermic comfort was recognized as a critical issue for the comfort of the exoskeleton, both for those supporting the back (60) and those supporting the upper arms (57, 58, 65), to the point that it could inhibit the use of the device (65). In an automotive assembly, after 3 months of regular use of an upper limb supporting exoskeleton, participants reported a substantial decrease in the amount of discomfort experienced in their neck and arms and a slight reduction in back discomfort (65). For press and shear workers, higher significant discomfort scores wearing the back support exoskeleton were found located at the chest and thighs, compared to those not wearing the exoskeleton and the discomfort scores were much higher as experienced by study participants in a lab situation (46). In order for picking activities and logistic activities, the support at the legs and the hip belt was two determinants of the comfort of the back support exoskeleton; workers reported that it would be not possible to wear the exoskeleton during the whole day (60). Carrying heavy loads across the facilities was associated with perceived discomfort in the chest and thighs, showing the need for optimization of the back support exoskeleton (68). In care settings, several nurses complained about poor fit, referring that wearing the back support exoskeleton made them stiffer and unable to react to sudden situations (67). Finally, perceived comfort may widely vary between men and women: in Hwang et al. study (56), most participants are women and the different tropism and muscle reserves could impact the perception of comfort. Also, the interface between the back support exoskeleton and the chest in female workers can be a noticeable issue in terms of not only comfort but also usability and intention to use (48, 56).

### Perceived exertion and fatigue

Nine studies (39%) analyzed the exertion and fatigue perceived by workers wearing the device. The most used scale was the RPE scale, adopted by three studies. Overall, as for usability and comfort, it was found great heterogeneity in the methods used to measure exertion and fatigue. In plastering workers, the perceived exertion was reduced using the upper limb supporting exoskeleton, in particular for activities at the ceiling, while during tasks involving various movements passive exoskeletons were found not as effective as during less varied tasks (52). In the automotive assembly setting, using an exoskeleton had little impact on perceived work intensity or musculoskeletal disturbs. The beneficial effects did not occur immediately and were observed after a 6-month period of continuous use. However, the reduction of physical demand on the shoulder, neck, and back was perceived positively by workers wearing the upper limbs exoskeletons (57, 58). Nevertheless, a portion of the reduced exertion perceived in the shoulders and the arms could be attributed to a sort of placebo effect elicited by users' positive expectations (63). Upper limbs exoskeletons were judged effective in increasing endurance time and reducing perceived effort while holding demanding postures with raised arms and/or having to lift and hold small work tools (66). Reducing workload, however, may not be always linked to good usability, as proved by logistic workers wearing shoulders supporting exoskeletons who experienced a reduced temporal workload, but still scored the usability moderate (51). Testing the upper limbs exoskeletons in the surgical room, the assistive device is perceived by workers to be able to address the high perceived physical workload among residents, who often maintain static posture, such as when holding instruments (e.g., scopes or retractors) (48). In general, subjects experienced significantly less pain in their shoulders after 1 day of operating with the exosuit compared to 1 day of operating without the exosuit. Subjects also reported not only decreased neck, upper arm, wrist, and knee pain but also a small increase in lower back pain after wearing the device (59). In an enclosure production line, participants reported higher exertion scores in the in-field session compared to the simulated session (63). In wasting collecting activity, results showed that the perceived exertion of the workers using the Ergo-Vest was significantly lower than those who did not use the device; the work difficulty perceived wearing the exoskeleton was one level lower than without the device (69).

### Occupational safety and health

Only three studies addressed the issue of improvement of the participant's health and prevention of injuries, and one study considered the possible harmfulness of the exoskeleton regarding safety in the workplace. In an 18-month experiment, automotive operators reported an equivalent or slightly better perceived safety and slightly better perceived performance. Those who used the ASE were roughly half as likely to make a medical visit that involved an injury to or pain in the upper extremity (58). In the three participants of the study of Smets (65) who had varying levels of musculoskeletal discomfort (shoulder, neck, and back), a decreasing trend was observed in the total musculoskeletal disturbs and at the end of the 3 months of regular exoskeleton use, the scores were zero for all three. In terms of self-perception, the perception of safety and health varies across the workers: some participants described the benefits of exoskeleton use in terms of feeling more support or facilitating better posture; on the contrary, others who described having a strong or healthy back did not perceive these long-term health benefits (62). Finally, in particular working environments such as nurse homes, workers were concerned about their safety because the patients, mostly affected by dementia, could grab onto the device possibly causing injuries (67).

### Job performance and productivity

Eight (33%) studies considered the topic of job performance and productivity. The efficacy of the exoskeleton widely varies according to the task performed. In the automotive setting, tasks involving heavy material handling were reported to be easier to perform with the exoskeleton, while more dynamic tasks which involved various postures were classified as harder to perform, and sometimes the exoskeleton might disturb rather than help. Also, dynamic tasks, which require adjustments in the exoskeleton support, were difficult to manage due to time pressure (53). Exoskeletons were reported to improve endurance and accuracy execution of precision tasks (66). In a 3-month experience, a slight gain in self-reported task performance was observed; participants reported that they received the most benefit from the exoskeleton when performing tasks that were overhead, while they reported continued difficulties with tasks that required even moderate nonneutral trunk postures (65). Plasterer workers reported the risk of hindrance and slowness of the productivity process (52), and a similar finding was retrieved in the study by Omoniyi et al. (62), in which some farmers reported being able to accomplish more shoveling work faster with less energy, while others felt encumbered by tension in leg pads while walking. Using the exoskeleton while driving or operating farm machinery was often described as an encumbrance and did not provide appropriate support. Also in a logistic facility, some workers felt constrained rather than relieved by the exoskeleton, or that its functions did not correspond well enough with their work tasks to be considered useful (64). While the exoskeleton provided a positive effect in lifting and lowering loads, when carrying the load and walking without it, the suitability of the exoskeleton is greatly reduced (63). On the contrary, the exoskeleton model tested in the waste-collecting activities did not create a restriction on workers' motion (69).

### Acceptability and intention to use

Twelve studies (50%) dealt with the acceptability and the intention to use exoskeletons by workers. Fit and comfort are in general key determinants of the possible adoption of the exoskeleton by workers (57, 58, 65), along with perceived performance (58). Also, a perceived decrease in physical demands when using the exoskeleton was positively associated with reported intention-to-use. Nevertheless, intention-to-use was strictly connected to usability and negatively impacted by discomfort, to the extent that even a minimal level of discomfort might hinder a user's acceptance (55). At the same time, it was pointed out that workers tended to judge more positively the exoskeleton *per se* for its characteristics and potential than its use in a real field social aspects come into play, linked to how others may judge an individual who decides to use the device or how the person perceives the decision and the imposition of the exoskeleton (54). In a study involving enclosure workers, although the exoskeleton obtained good levels of usability and acceptability, lower scores were observed on aspects related to image and output quality. Workers also identified major improvements to perform before considering to use constantly the device at work (52, 61). Among surgical team members, workers answered positively when asked whether they would be frequently using this device (48) and most of them would consider incorporating the exosuit into their daily practice (59). The aspects of attractiveness, perspicuity, efficiency, dependability, and stimulation were judged neutral by shear workers, while the exoskeleton was judged positively about the novelty (46). In the automotive assembly setting, user acceptance was found to decrease significantly by the end of the period of exoskeleton use (4 weeks), after being rated very high at the beginning (55). The exoskeleton acceptance was found associated with technology-induced self-efficacy beliefs, which are in turn moderated by exertion relief capacity and usefulness attributed to the device (64). In a geriatric care setting (67), the intention to use the exoskeleton was associated with its perceived usefulness and how enjoyable it was to use. Besides, the exoskeleton's trustworthiness, inked to personal and procedural characteristics of technological use, was a possible determinant of the intention to use.

## Discussion

This systematic review included 24 studies, in which the exoskeletons were tested on workers performing real occupational tasks, both in the lab setting and directly in the field. Despite the online research being performed without any date restriction, all the studies were published in the last five years, mostly in 2020 and 2021. This testifies to the novelty of the introduction of industrial exoskeletons in real working settings.

### Models of exoskeletons tested in the selected studies

All the papers included in our review used passive exoskeletons which use the restoring forces of springs, dampers, or other materials to support the human movement. The movements of the user generate the energy stored in a passive exoskeleton, and forces are redistributed to protect specific body regions. The improvement of the performance of the exoskeleton user is not given by additional physical strength (as it would be for active devices) but by the ability provided by passive exoskeletons to maintain exhausting positions over a longer period of time (70). Passive exoskeletons are relatively light (from 1 to 5 kg approximately in our selection) and have an affordable cost. Also, there are several models already available on the market and overall require acceptable maintenance costs. The exoskeletons tested in the selected studies showed good usability and acceptable comfort; almost all the models are already in the market, so they can be considered in an advanced development phase. Although a lack of intervention effectiveness studies in the field, industrial exoskeletons are already being used in several work settings, as also testified by the heterogeneity of the job included in our review. Active exoskeletons instead are still very limited on market, due to the higher costs and the need for further technological developments. With the purpose of collecting evidence only on exoskeletons used in real occupational fields, this review excluded studies addressing active devices. Indeed, we found only one study providing an on-field experience of an active device (85), which was tested among military personnel and determined increased workload perceptions among participants. Also in lab experiments, often the active exoskeleton is tested only on very few participants, limiting the quality of evidence (70). According to Toxiri et al. (86), active exoskeletons offer greater versatility and are potentially suited to provide stronger assistance. Active devices may, therefore, be more appropriate for demanding and dynamic tasks, such as handling heavier loads. This was also found by a recent study comparing active and passive back exoskeletons in the lab, showing that in dynamic conditions, the active device had better scoring (87). Active devices may have a better potential in reducing physical load than passive ones, but they are much heavier, with the lighter ones weighing from 6 to 9 kg (88, 89), and can provide higher pressure at the body–machine interface (90).

### Exoskeletons and WMSDs

Previous studies and reviews have focused on the effects of exertion and reduction in muscle activity provided by industrial exoskeletons, mainly through lab experiments. Only recently, since the beginning of the use of exoskeletons in working settings, user experience has gained relevance in research. Currently, prospective studies analyzing the intervention of using exoskeletons to reduce the incidence of WMDS are still missing. Industrial exoskeletons were born and marketed with the aim of reducing WMSDs, assuming that the reduction in exertion, which is the outcome most analyzed in the current literature, corresponds to a reduction in musculoskeletal disease risk factors. WRMSDs have a complex pathogenesis (91), and such an assumption may appear too simplistic. It is nevertheless true that if exoskeletons achieved reductions in the mechanical stressors associated with manual handling tasks, they have the potential to reduce the incidence of WMSDs and the related burden of disease, including the economic cost of management of working days loss and disability (92, 93). In our review, only the study of Kim et al. (57) provided some evidence of the reduction of medical visits requested by workers wearing the exoskeleton for a long period of time. Certainly, further research, involving a long period of observation and medical records, is needed in this field. As pointed out by the consensus guidelines by Steinhilber et al. (94), the use of exoskeletons should be monitored medically by the occupational physician, with regular interviews and medical examinations. Longitudinal studies and case-control studies are needed to evaluate the effects of industrial exoskeletons on workers' health.

### Safety and risk assessment for exoskeletons in occupational settings

There are several different opinions on how industrial exoskeletons might be considered. They can be defined as Personal Protective Equipment (95), or as performance and amplification devices (PAADs). As PPE, they should be considered for the health protection of workers only when all other organizational measures to reduce occupational risks are taken and certified according to the Regulation (EU) 2016/425 on personal protective equipment (96). However, there are many workplaces that are not tied to a specific location (e.g., agriculture), where ergonomic design measures cannot be implemented because of the changing environmental requirements and where heavy manual material handling is common and injury risk is high (97). As PAADs should be considered during the workplace risk assessment, the potential risks of exoskeletons in working environments, for example, during a slip or a fall accident and perhaps the massive introduction of exoskeletons in industrial settings should pass through a redesign of workplaces (98). According to our findings, in some cases, workers have recognized exoskeletons as a hindrance and that can represent an issue for security, for example, in case of workplace evacuation during an emergency. For this reason, the donning and the doffing of the device are crucial points when considering usability, and therefore, the intention to use the exoskeleton in real settings. Moreover, some authors suggested that exoskeletons should not be used to motivate increased work demands or duration, since the levels of activity observed in secondary muscle groups other than the target area are largely unchanged (99), and no change in the overall physical demand of the task is observed (100).

As the introduction of exoskeletons is becoming more and more frequent in industrial settings, studies designed to study the potential risks linked to exoskeletons are needed (93). In our selection, there was no evidence of any specific risks to the health and safety of workers, but the findings underlined that the exoskeleton is not useful in dynamic tasks but is considered by workers as a hindrance. This consideration leads to the need to accurately select the task in which wearing the exoskeleton, as pointed out also by lab studies (101).

### The difference between lab and field studies

In the field, studies show overall less brilliant results than lab studies, in terms of user satisfaction. The work tasks are far more complex than the experimental tests and exoskeletons are the most useful during static postures, for example, when it is necessary to keep the arms above the shoulders for a long period of time (e.g., assembly line overhead). The differences in the performed tasks, with those in real working environments far more complex than those studied in the lab, is the first great difference between lab and field studies. Recent research has also pointed out how wearing an exoskeleton and performing complex tasks impose greater motor adaptation and neurocognitive efforts, which may almost offset the biomechanical advantages of exoskeletons (102). Industrial exoskeletons can be efficient in physical exertion reduction but not for all tasks and not for all workers, because they cannot be considered one-size technology. In our review, the working population tested was mainly constituted of healthy male subjects and for the most part, one criterion of exclusion from the experiment was having any musculoskeletal disturbs. Only a study included a very restricted sample of workers with some low back disturbs (65) who definitely benefit from wearing the exoskeleton during work. In this regard, even if the studies tested a working population, they could not be considered fully representative, due to the high prevalence of WRMSDs in Europe (1). The two studies including female workers – whose number is currently growing in the workplace – evidenced issues about the adaptation of the device to the shape of the female body. Differences in kinematics wearing exoskeletons between men and women were already found, but it is still unknown if they can have a relevant meaning (99).

### Usability and acceptance by real workers

Comfort and fit indeed are the main factor influencing the usability and acceptance of the device by workers. Workers may accept the exoskeleton only if the benefits perceived will overcome the possible negative residual drawbacks, like the overall discomfort (heath, human-machine interface, time to don and doff) (103). Despite the heterogeneity of the jobs analyzed and the exoskeletons tested, the wearability and the easiness of taking on and off the exoskeleton remain key factors for the usability and intention-to-use of the device. An interesting point is that when the measure of subjective experience was repeated over time, it decreased after a first enthusiastic evaluation. This was consistent with a previous finding in a lab test where subjective ratings of perceived discomfort and usability worsened during the time (104). The novelty and the enthusiasm of wearing a novel device can be a driving force for workers, which are obviously destined to decrease over time so longitudinal evaluations are necessary to discriminate it and to obtain a not-biased evaluation that can also provide a suggestion for further development. Overall, the findings of our review underline the need for a human-centered design (103) that aims at making systems usable and useful by focusing on the users, their needs, and their requirements, and by applying human factors/ergonomics, and usability knowledge and techniques. In this optic, the development and the large-scale adoption of industrial exoskeletons can be contextualized in the framework of Industry 5.0 whose main concern is the synergy between humans and machines (105).

### Limitations and strengths of the evidence retrieved

This review has several limitations and points of strength. First, the evidence retrieved is subject to the quality of the articles included in the review. The selected papers have a cross-sectional design for the most part, and in some cases adopted a qualitative approach, and this impacts the level of confidence of the evidence. The lack of standards in evaluating the outcomes and the use of not-validated questionnaires makes it hard to compare studies and generalize the findings. Second, as mentioned above, the participants of the studies and the occupational settings involved may not be fully representative of the general working population and working environments in general. Regarding the methodology adopted in this systematic review, although performed according to PRISMA guidelines and with a rigorous search and selection strategy, it is possible relevant studies may have been missed. However, to the best of our knowledge, this is the first review that systematically addressed on-field studies involving real workers wearing exoskeletons and analyzing the depth of their user experience. It identified major points to consider when introducing exoskeletons on the field, such as comfort, job performance, acceptance from workers, task specificity, safety, and health issues.

## Conclusion

The use of exoskeletons in occupational settings is a relatively new phenomenon with a limited amount of research available on the topic. A systematic review of the literature was conducted in order to identify and evaluate the available evidence on the field use of exoskeletons from an occupational safety and health perspective. Twenty-four scientific articles were identified that met the inclusion criteria for the review. The findings of the review suggest that exoskeletons have the potential to reduce the risk of musculoskeletal injuries in a variety of occupational settings. However, there is a lack of evidence on the long-term safety and efficacy of exoskeleton use in the workplace. Additionally, there are several potential concerns that need to be addressed, such as safety issues and ergonomics, considering that is not clear if the exoskeleton itself can cause the discomfort, or if the discomfort should be due to the lifting tasks that the workers are called to perform. More research is needed to determine the most effective and safe ways to implement exoskeleton use in occupational settings.

Our review explored the experience of real workers wearing industrial exoskeletons. The lack of longitudinal studies is the core limitation when analyzing such data and medical data regarding the possible prevention of WMDs using exoskeletons are currently missing. On-field studies, which addressed workers' experience wearing exoskeletons, showed overall less brilliant results than lab experiments, due to the higher complexity of work tasks compared to stereotyped exercises. Exoskeletons are not a fix-all technology, neither for workers nor for job tasks; they tend to show more of their potential in static activities while in dynamic tasks they can obstacle regular job performance. Comfort and easiness of use are the key factors influencing the user's experience.

The existing literature on the field use of exoskeletons is mostly anecdotal, consisting of case reports and small case series. There is a need for larger, well-designed studies on the field use of exoskeletons in order to better understand the potential safety and health risks associated with their use.

## Author contributions

The first draft, conceptualization, and the original idea were provided by AB and LGL. The literature search was performed by LGL and AM. The selection of articles was performed by LGL, AM, and AB. The study was critically revised and reviewed by NM, GA, FC, and LF. All authors contributed to the article and approved the submitted version.

## Conflict of interest

The authors declare that the research was conducted in the absence of any commercial or financial relationships that could be construed as a potential conflict of interest.

## Publisher's note

All claims expressed in this article are solely those of the authors and do not necessarily represent those of their affiliated organizations, or those of the publisher, the editors and the reviewers. Any product that may be evaluated in this article, or claim that may be made by its manufacturer, is not guaranteed or endorsed by the publisher.
