# Supplementary material for: Industrial exoskeletons from bench to field: Human-machine interface and user experience in occupational settings and tasks
Source: Front Public Health. 2022 Nov 21;10:1039680. doi: 10.3389/fpubh.2022.1039680 (PMC9720272; doi:10.3389/fpubh.2022.1039680)
Supplement: Supplementary file 1 [file Table_1.DOCX]

**Industrial exoskeletons from bench to field: Human Machine Interface and User Experience in occupational settings and tasks**

International archives of occupational and environmental health

A. Baldassarre^1^, L.G. Lulli^1^, F. Cavallo^2^, N. Mucci^1^, L. Fiorini^2^, A. Mariniello^3^, G. Arcangeli^1^

1. Department of Experimental and Clinical Medicine, University of Florence, Largo Brambilla, 3, 50134 Florence, Italy
2. KKK
3. School of Occupational Medicine, University of Florence, Largo Brambilla, 3, 50134 Florence, Italy.

**Corresponding Author: Lucrezia Ginevra Lulli, lucreziaginevra.lulli@unifi.it**

**Literature search strategy for the systematic review**

1. Search strategy for MEDLINE (PubMed), run on 20th October 2020 and updated through rerunning on June 3th 2021.

*exoskeleton* OR wearable robotic system*) AND (occupational medicine OR occupational health OR occupational safety OR occupational health and safety OR preventive medicine OR ergonomics OR musculoskeletal disease* OR musculoskeletal disorder* OR workload* OR work related disease* OR work related illness* OR work-related disease* OR work-related illness*)*

1. Search strategy for EMBASE. The search was performed on June 3th 2021 using the PICO search tool, updated on August 23th 2021.

*('worker'/exp OR 'laborer' OR 'labourer' OR 'worker') AND ('exoskeleton'/exp OR 'exoskeleton'')*

1. Search strategy for Web of Science. The search was performed on June 4th 2021 and updated on August 27th 2021.

*(ALL=((WORKER) AND ((EXOSKELETON) OR (WEARABLE ROBOTIC SYSTEM)))) AND ALL=((COMPLIANCE) OR (USABILITY) OR (PERCEPTION))*

1. Search strategy for Scopus. The search was performed on 27th August 2021.

## *TITLE-ABS-KEY ( ( worker* ) OR ( occupational ) ) AND TITLE-ABS-KEY ( exoskeleton ) AND TITLE-ABS-KEY ( ( perception ) OR ( usability ) OR ( compliance ) OR ( subjective ) )*
